# Homologous recombination repair rathway and RAD54L in early-stage lung adenocarcinoma

**DOI:** 10.7717/peerj.10680

**Published:** 2021-02-16

**Authors:** Shaopeng Zheng, Lintong Yao, Fasheng Li, Luyu Huang, Yunfang Yu, Zenan Lin, Hao Li, Jin Xia, Michael Lanuti, Haiyu Zhou

**Affiliations:** 1 Department of Thoracic Surgery, Cancer Hospital of Shantou University Medical College, Shantou, Guangdong, P.R. China; 2 Division of Thoracic Surgery, Guangdong Provincial People’s Hospital & Guangdong Academy of Medical Sciences, School of Medicine, South China University of Technology, Guangzhou, P.R. China; 3 Shantou University Medical College, Shantou, P.R. China; 4 The Second School of Clinical Medicine, Southern Medical University, Guangzhou, P.R. China; 5 Guangdong Provincial Key Laboratory of Malignant Tumor Epigenetics and Gene Regulation, Department of Medical Oncology, Phase I Clinical Trial Centre, Sun Yat-sen Memorial Hospital, Sun Yat-sen University, Guangzhou, P.R. China; 6 Graduate School, Guangzhou University of Chinese Medicine, Guangzhou, P.R. China; 7 Department of Surgery, Division of Thoracic Surgery, Massachusetts General Hospital, Boston, MA, USA

**Keywords:** Lung adenocarcinoma, Homologous recombination repair, RAD54L, Prognostic factor

## Abstract

**Objective:**

The current study aims to identify the dysregulated pathway involved in carcinogenesis and the essential survival-related dysregulated genes among this pathway in the early stage of lung adenocarcinoma (LUAD).

**Patients and Methods:**

Data from The Cancer Genome Atlas (TCGA) including 526 tumor tissues of LUAD and 59 healthy lung tissues were analyzed to gain differentially expressed genes (DEGs). Gene ontology (GO) analysis was conducted with DAVID, while the Kyoto Encyclopedia of Genes and Genomes (KEGG) pathway analysis of DEGs was performed, followed by gene set enrichment analysis (GSEA) methods. Survival analysis was implemented in TCGA dataset and validated in Gene Expression Omnibus (GEO) cohort GSE50081, which includes 127 patients with stage I LUAD.

**Results:**

GSEA enrichment analysis suggested that homologous recombination repair (HRR) pathway was significantly enriched. Subsequent KEGG pathway enrichment analysis indicated the significant up-regulation of HRR pathway in patients with T1 stage LUAD. Retrieved in Gene database, RAD54L is involved in HRR pathway and were recognized to be significantly differentially expressed in T1 stage LUAD in our study. The survival analysis indicated that high expression of RAD54L was significantly related to worse overall survival in patients with T1 stage LUAD (TCGA cohort: HR=2.10, 95% CI [1.47–2.98], *P* = 0.001; GSE50081 validation cohort: HR = 2.61, 95% CI [1.51–4.52], *P* = 0.002). Multivariate cox regression analysis indicated that RAD54L is an independent prognostic factor in the early-stage LUAD.

**Conclusion:**

HRR pathway is up-regulated in LUAD, among which the expression of RAD54L was found to be significantly differentially expressed in T1 stage tumor tissue. Patients with high expression of RAD54L were associated with worse overall survival in the TCGA cohort and validation cohort. This study suggests a potential mechanism of lung cancer progression and provide a budding prognostic factor and treatment target in early-stage LUAD.

## Introduction

Lung cancer has the leading mortality rates worldwide, accounting for 12.7% in all new cancer cases (Siegel, Miller & Jemal, 2020). Among all subtypes, lung adenocarcinoma (LUAD) generates from glandular and mucosal glands in alveoli or small airways and occupies 40–50% of lung cancer (Miyahara et al., 2019).

Surgery resection is the most effective management in early-stage LUAD, while the overall survival and recurrence remains poor (Liu et al., 2020). Choices of comprehensive therapeutic strategies depend on TNM staging, which neglects the genetic and molecular characteristics of cancer cell. It is imperative to discover genetic biomarkers in early-stage LUAD to integrate with TNM staging and stratify the poor prognosis and recurrence patients.

During the carcinogenesis of LUAD in early stage, genomic instability and accumulating oxidative stress from exogenous or endogenous gene toxin cause DNA damage, which provoke the progression of lung cancer and is offset by DNA damage and repair (DDR) system (Caramori et al., 2019; Sears, 2019; Jamal-Hanjani et al., 2017). DDR system deploys various repertoire of mechanism to preserve genetic integrity and alleviates the DNA disturbance by double or single strand DNA repair. Previous studies found that impairment or disruption of DDR system and the deriving genomic instability was associated with carcinogenesis and tumor progression (Lee et al., 2016; Greulich & Cherniack, 2019; Burrell et al., 2013).

Base on the tumorigenesis function of DDR system, we hypothesize that dysregulation of DDR system promotes the tumor progression in early-stage LUAD and functional genes in DDR system could be potential prognostic factors for early-stage LUAD. This study attempts to identify the significant dysregulated genes and pathways in early progression of LUAD with clinical impaction.

## Materials and Methods

### Patients and sample

**Figure 1:**
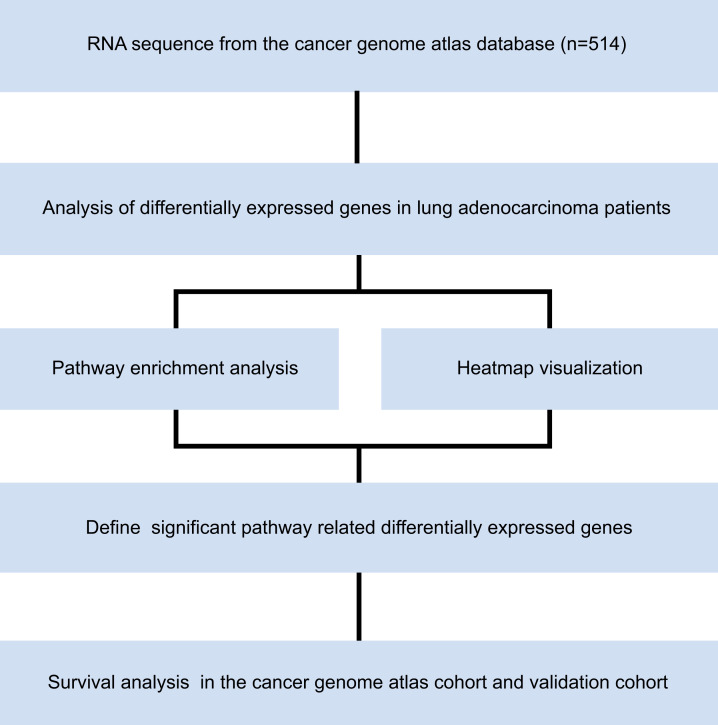
Study flow chart.

RNA sequences from 514 patients with LUAD in The Cancer Genome Atlas (TCGA) were downloaded from the University of California Santa Cruz (UCSC) Xena database (TCGA hub; LUAD), which were sequenced in the IlluminaHiSeq platform. This dataset composes of 526 tumor tissues and 59 healthy lung tissues. Microarray dataset of GSE50081 was downloaded from Gene Expression Omnibus (GEO) database. We established validation cohort by 127 patients with stage I LUAD in GSE50081. Pathological tumor stage was evaluated according to American Joint Committee on Cancer (AJCC) Cancer Staging Manual, eighth edition. The study was conducted from April 1st, 2019, to May 1st, 2020, in accordance with the principles stated in the Declaration of Helsinki. The overall study design is shown in Fig. 1. The requirement of informed consent was waived by the ethics committee because all the data were obtained from open access public database.

### Identification of differentially expressed genes and functional enrichment analysis in lung adenocarcinoma

To impute the possible data missing at random, which is ubiquitous in various dataset, gene data was imputed with impute package, providing unbiased and valid data for assessment. Differentially expressed genes (DEGs) between tumor tissues and healthy tissues are identified, which with highest *P* value are visualized in heatmap. Gene ontology (GO) enrichment analysis was performed by DAVID website (https://david.ncifcrf.gov/) to annotate the gene in terms of biological processes (BP), molecular functions (MF), and cellular components (CC), demonstrating the molecular and cellular biology functions of DEGs.

### Identification of differentially expressed genes and functional pathway enrichment analysis in T1 stage lung adenocarcinoma

To explore prognostic genes from DDR systems in early-stage LUAD, we further identified the DEGs in T1 stage, followed with gene and pathway enrichment analysis. Uploading the expression data of total normalized mRNAs to gene set enrichment analysis (GSEA) v3.0 software, GSEA was conducted based on Kyoto Encyclopedia of Genes and Genome (KEGG) database. KEGG pathway enrichment analysis was performed subsequently to investigate significant functional pathways among differentially expressed genes.

### Survival analysis and validation of significant pathway related differentially expressed genes

Through DEGs analysis, we aim to find out significant pathways and their critical related genes. The expression of crucial DEGs in different tumor stages was evaluated and compared. Survival analysis of those crucial DEGs is conducted by Kaplan–Meier analysis and validated in GSE50081 cohort. Prognostic factors were investigated by univariate cox regression analysis among crucial DEGs and clinical characteristics in the TCGA cohort and GSE50081 cohort. All significant factors were applied into the multivariate cox regression analysis to further identify independent prognostic factors.

### Statistical analysis

Differentially expressed genes are extracted by limma package with the standard of the absolute value of log_2_ fold change more than 1, and the adjusted *P* value less than 0.05. Benjamini–Hochberg false discovery rate method was applied to adjust *P* value and decrease the false positive rate. The pheatmap package presented results of DEGs identification by hierarchical clustering analysis. The enrichment function in terms of BP, MF, CC was visualized by GO analysis with GOplot package. The enrichment pathway of KEGG analysis was visualized in dotplot, which were constructed by ggplot2, grid, devtools and easygplot2 packages. The GSEA results were exhibited by the GSEA enrichment plot. The comparison among expressions of significant DEGs in different tumor stages was conducted by Kruskal−Wallis test. Survival analysis was conducted by survminer package and the significance was calculated by log rank test, followed with cox proportional hazards regression model to calculate hazard ratio and confidence interval. All the package above are conducted based on R software. Online tools like Gene Expression Profiling Interactive Analysis (http://gepia.cancer-pku.cn/) and Kaplan-Meier plotter (https://kmplot.com/analysis/index.php?p=service&cancer=lung) are applied to validate the survival analysis (Tang et al., 2017; Gyorffy et al., 2013).

## Results

### Participants

In this study, 514 patients with LUAD comprise TCGA cohort (female: 53.50%; median (IQR) age: 66 (59–72)). The validation cohort GSE50081 from GEO database included 127 patients with LUAD (female: 48.82%, median (IQR) age: 69.92 (62.79–75.69)).

Our study tends to explore significant pathways and genes in early-stage LUAD. According to pathological TNM stage, 53.70% (276/514) patients were diagnosed with stage I LUAD and 23.54% (121/514) patients were diagnosed with stage II LUAD in TCGA cohort. In the GSE50081 cohort, 72.44% (92/127) patients were diagnosed with stage I LUAD and 27.56% (35/127) patients were diagnosed with stage II LUAD. Detail clinical characteristics are shown in Table 1.

**Table 1:** Clinical characteristics of the TCGA cohort and GSE50081 cohort.

Characteristics	TCGA (*n* = 514)	GSE50081 (*n* = 127)
Age (years)		
Median (IQR)	66 (59–72.5)	69.92 (62.79–75.69)
≤65y, No. (%)	238 (46.30%)	40 (31.50%)
>65y, No. (%)	257 (50.00%)	87 (68.50%)
Missing	19 (3.70%)	0 (0%)
Gender, No. (%)		
Female	275 (53.50%)	62 (48.82%)
Male	239 (46.50%)	65 (51.18%)
pT stage, No. (%)		
T1	169 (32.88%)	43 (33.86%)
T2	276 (53.70%)	82 (64.57%)
T3	47 (9.14%)	2 (1.57%)
T4	19 (3.70%)	0 (0%)
Missing	3 (0.58%)	0 (0%)
pN stage, No. (%)		
N0	338 (65.76%)	94 (74.02%)
N1	95 (18.48%)	33 (25.98%)
N2	74 (14.40%)	0 (0%)
N3	2 (0.39%)	0 (0%)
Missing	5 (0.97%)	0 (0%)
pM stage, No. (%)		
M0	486 (94.55%)	127 (100%)
M1	27 (5.25%)	0 (0%)
Missing	1 (0.19%)	0 (0%)
pTNM stage, No. (%)		
Stage I	276 (53.70%)	92 (72.44%)
Stage II	121 (23.54%)	35 (27.56%)
Stage III	84 (16.34%)	0 (0%)
Stage IV	26 (5.06%)	0 (0%)
Missing	7 (1.36%)	0 (0%)

**Note:**

TCGA, The Cancer Genome Atlas; pT stage, Pathologic T Stage; pN stage, Pathologic N Stage; pM stage, Pathologic M Stage; pTNM stage, Pathologic TNM Stage.

### Identification of differentially expressed genes and functional enrichment analysis in lung adenocarcinoma

**Figure 2:**
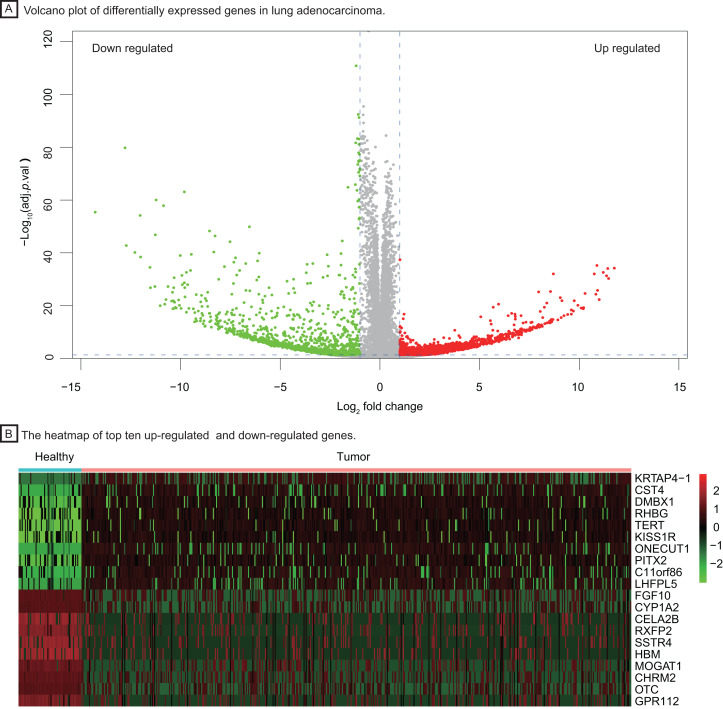
Identification of differentially expressed genes between healthy lung tissues and tumor tissues. (A) Volcano plot of differentially expressed genes in lung adenocarcinoma. (B) The heatmap of top ten up-regulated (red) and down-regulated (green) genes.

To investigate important dysregulated genes and their functions in LUAD, we performed a DEG analysis between 526 tumor tissues of LUAD and 59 healthy lung tissues. Differentially expressed genes were screened out according to the log_2_ fold change and adjusted *P* value (|log_2_ fold change| > 1, *P* < 0.05, Fig. 2A), obtaining 3,786 up-regulated and down-regulated genes in tumor tissue. The heatmap of the top ten up-regulated (red) and down-regulated (green) genes with the highest *P* value were shown in Fig. 2B.

**Figure 3:**
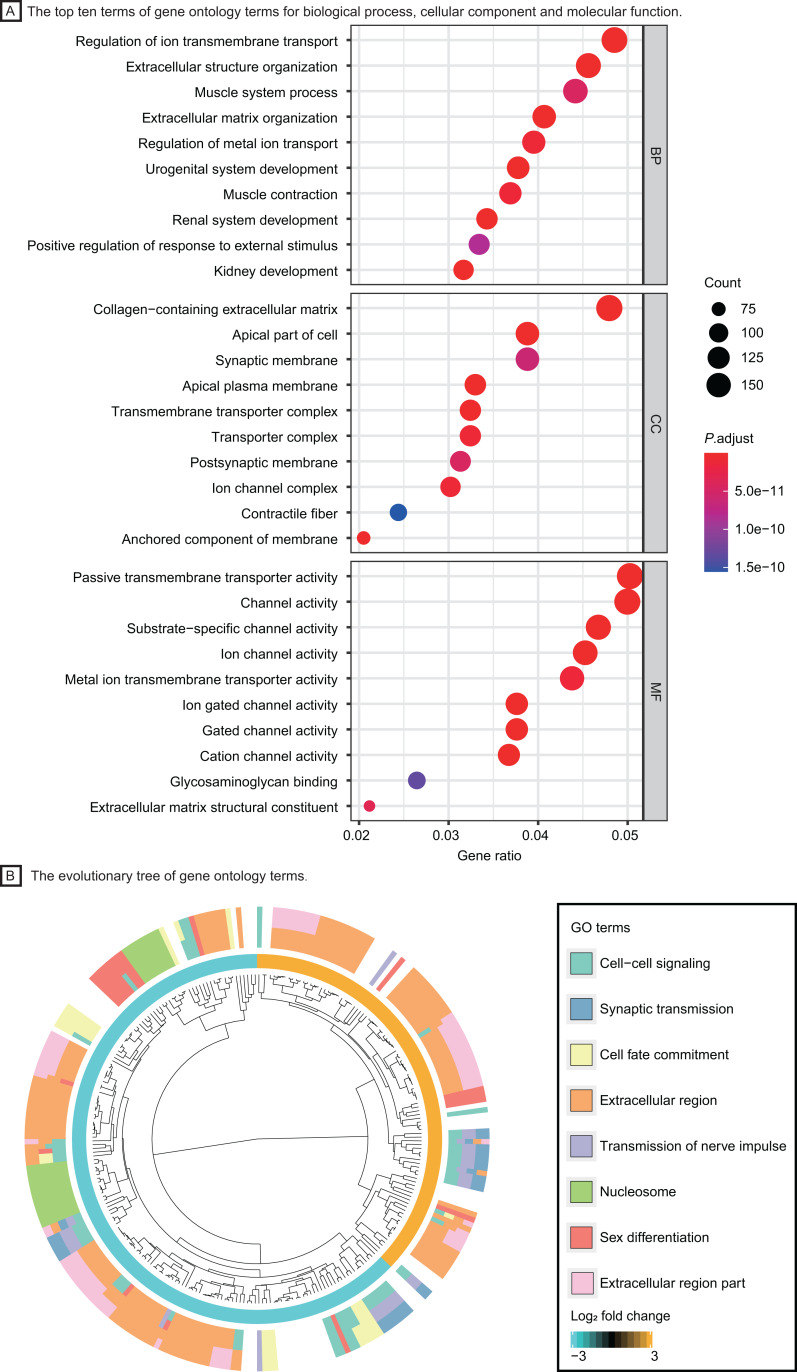
Functional enrichment analysis of LUAD patients. (A) The top ten terms of gene ontology terms for biological process, cellular component and molecular function. (B) The evolutionary tree of gene ontology terms. Abbreviations: BP, Biological Process; CC, Cellular Component; MF, Molecular Function; GO, Gene Ontology.

Based on the GO enrichment analysis in DAVID, DEGs were significantly enriched in regulation of ion transmembrane transport, extracellular structure organization for biological process, collagen−containing extracellular matrix, transmembrane transporter complex for cell component and passive transmembrane transporter activity, channel activity for molecular function. The top ten GO terms with highest P value were shown in Fig. 3A. The evolutionary tree (Fig. 3B) indicated that quite a few genes were differentially expressed with several GO terms, such as the nucleosome, extracellular region and cell-cell signaling, which may play an essential role in accelerating tumor proliferation, signal transmission and metastasis.

### Identification of differentially expressed genes and functional pathway enrichment analysis in T1 stage lung adenocarcinoma

**Figure 4:**
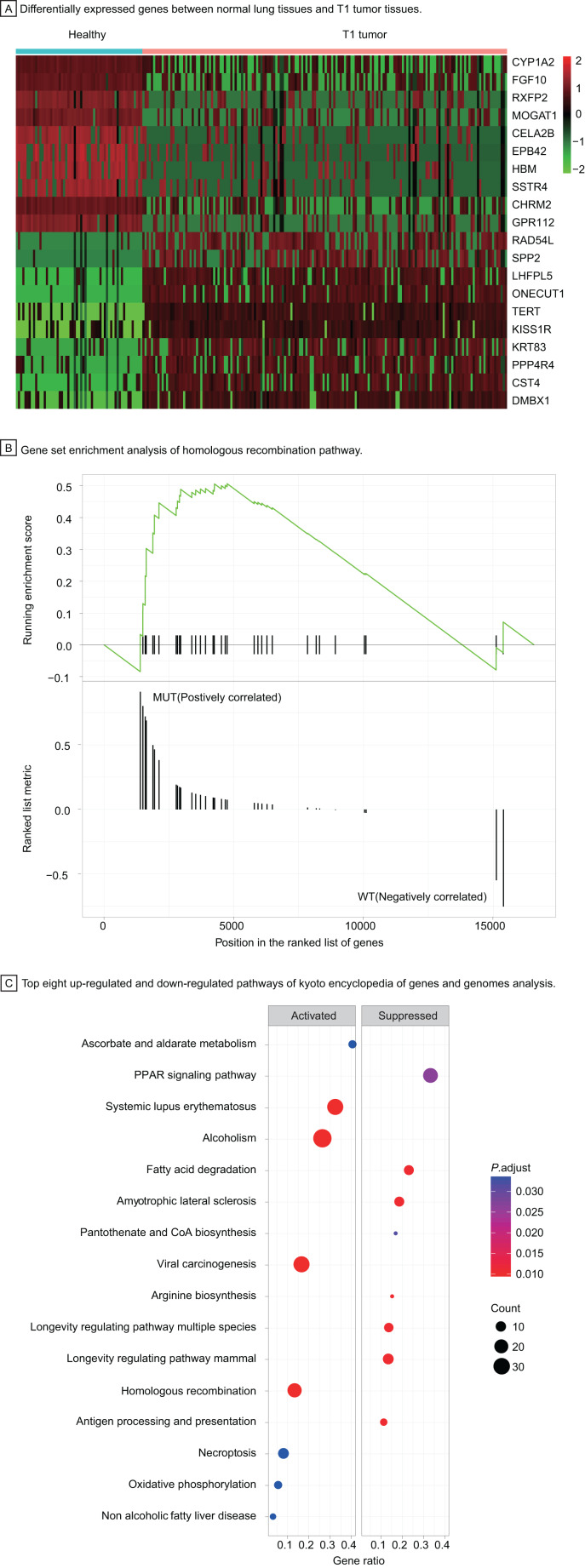
Identification of differentially expressed genes and functional analysis in T1 stage LUAD. (A) Differentially expressed genes between healthy lung tissues and T1 tumor tissues. (B) Gene set enrichment analysis of homologous recombination pathway. (C) Top eight up-regulated and down-regulated pathways of kyoto encyclopedia of genes and genomes analysis. Abbreviations: MUT, Mutation; WT, Wild Type; KEGG, Kyoto Encyclopedia of Genes and Genomes.

To understand the carcinogenesis mechanism and significant DEGs in early-stage LUAD, we analyzed gene expression of T1 stage tumors compared with that in healthy samples, identifying 3,447 differentially expressed genes with |log_2_ fold change| > 1, *P* < 0.05 (Fig. 4A). Most up-regulated DEGs were related to tumor invasion, metastasis, and progression. The HRR pathway was found to be significantly enriched in GSEA enrichment analysis (Fig. 4B). Up to 40 KEGG pathways were found to be enriched in T1 stage LUAD, of which pathways with top eight significance were displayed in dotplot (Fig. 4C).

Among up-regulated pathways in the KEGG analysis, the, most significant enrichment can be found in systemic lupus erythematosus, alcoholism, viral carcinogenesis, and homologous recombination repair (HRR) pathway. The HRR pathway, one of double-strand DNA repair mechanisms in DDR system, regulates the genetic recombination by exchanging nucleotide sequences between two similar or identical DNA strands to repair errors in replication. Hence, we explored further in the HRR pathway to investigate potential prognostic factors for early-stage LUAD.

### Identification of homologous recombination pathway related genes and survival analysis

**Figure 5:**
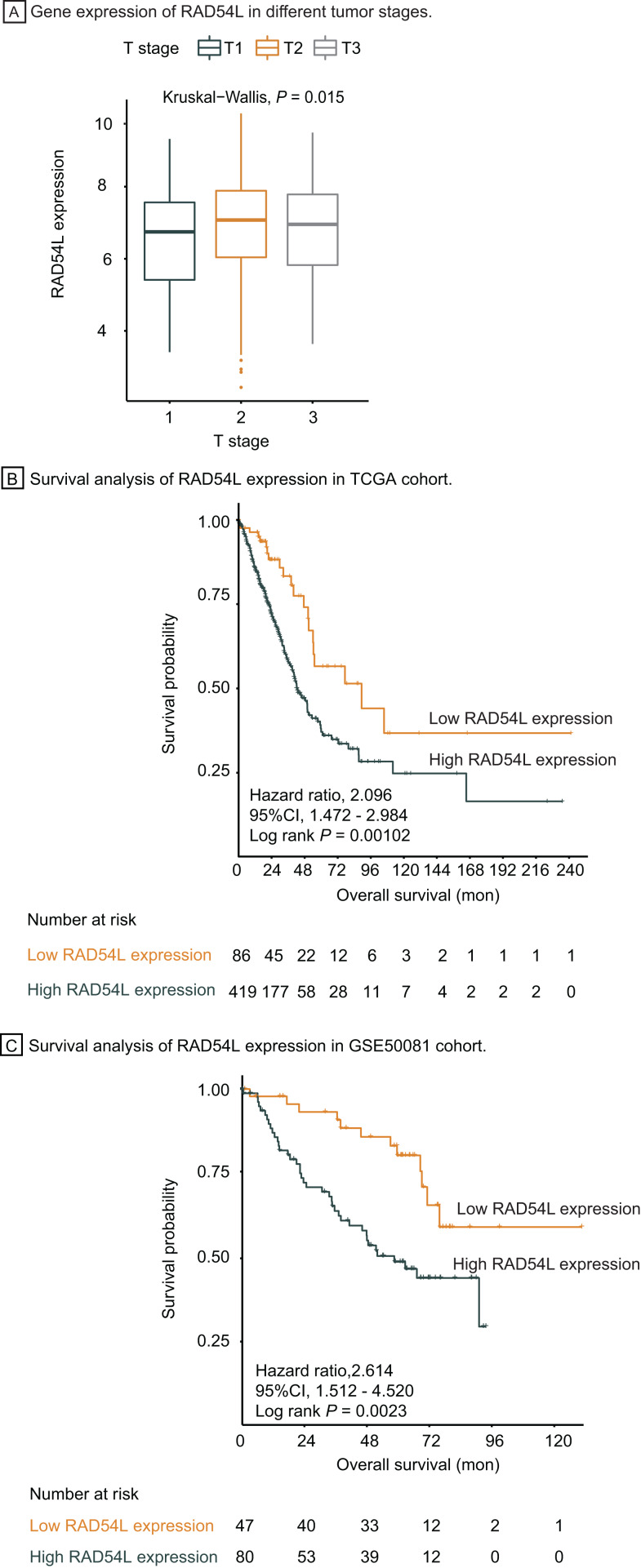
Survival analysis and validation of RAD54L. (A) Gene expression of RAD54L in different tumor stages. (B) Survival analysis of RAD54L expression in the TCGA cohort. (C) Survival analysis of RAD54L expression in the GSE50081 cohort. Abbreviations: TCGA, The Cancer Genome Atlas.

Genes involved in HRR pathway were retrieved, among which RAD54L was the most significantly DEGs in T1 stage LUAD. Gene expression of RAD54L in different tumor stage was shown in Fig. 5A, indicating that lower expression of RAD54L was detected in T1 stage comparing with T2 and T3 stage and the difference of expression among different T stage was significant (*P* = 0.015). Kaplan Meier analysis showed that the overall survival rate of patients with lower expression of RAD54L was significantly greater than that with higher expression of RAD54L (HR = 2.10, 95% CI: [1.47–2.98], *P* = 0.001) (Fig. 5B). Validation of survival analysis of RAD54L expression in GSE50081 cohort showed similar result that higher expression of RAD54L relates to worse overall survival (HR = 2.61, 95% CI [1.51–4.52], *P* = 0.002) (Fig. 5C). With online tools for survival analysis, GSE30219, GSE37745, GSE31210 and all 719 patients with LUAD were analyzed in Kaplan–Meier plotters, indicating that low expression of RAD54L associates with better survival (Fig. S1). Gene Expression Profiling Interactive Analysis confirmed these results as well (Fig. S2).

Univariate cox regression analysis of overall survival in TCGA cohort suggested that pathological T stage, pathological N stage, pathological M stage and expression of RAD54L were significantly correlated with OS, which were validated in the GSE500081 cohort and exported with identical result (Table 2). All significant factors above were incorporated into multivariate cox regression analysis, which indicated that pathological T stage, pathological N stage, pathological M stage and expression of RAD54L were independent prognostic factors for overall survival of LUAD patients in both TCGA and validation cohort (Table 3). RAD54L expression showed significant association with overall survival, with HR = 1.85, 95% CI [1.13–3.04], *P* = 0.014 in TCGA cohort and HR = 2.39, 95% CI [1.24–4.61], *P* = 0.009 in validation cohort, which implies that RAD54L is a potential prognostic factor and therapeutic target of early-stage LUAD.

**Table 2:** Univariate cox regression analysis of overall survival in the TCGA cohort and validation cohort.

Factor	TCGA cohort		GSE50081 cohort	
	HR (95% CI)	*P* value	HR (95% CI)	*P* value
Age (≤65 vs >65)	1.01 [0.99–1.02]	0.323	1.35 [0.73–2.50]	0.336
Gender (male vs female)	0.94 [0.72–1.29]	0.809	0.74 [0.43–1.29)	0.292
pT stage (T4 vs T3 vs T2 vs T1)	1.56 [1.30–1.87]	<0.001***	2.52 [1.34–4.76]	0.004**
pN stage (N3 vs N2 vs N1 vs N0)	1.73 [1.46–2.05]	<0.001***	2.08 [1.17–3.70]	0.013*
pM stage (M1 vs M0)	2.27 [1.35–3.79]	0.002**	NA	NA
RAD54L expression (high vs low)	2.11 [1.34–3.33]	0.001**	2.64 [1.38–5.04]	0.003**

**Notes:**

* *P* < 0.05.

** *P* < 0.01.

*** *P* < 0.001.

HR, Hazard Ratio; CI, Confidence Interval.

**Table 3:** Multivariate cox regression analysis of overall survival in the TCGA cohort and validation cohort.

Factor	TCGA cohort		GSE50081 cohort	
	HR (95% CI)	*P* value	HR (95% CI)	*P* value
Age (≤65 vs >65)	1.02 [1.00–1.03]	0.061	1.29 [0.69–2.39]	0.428
pT stage (T4 vs T3 vs T2 vs T1)	1.34 [1.11–1.63]	0.003**	2.06 [1.05–4.01]	0.035*
pN stage (N3 vs N2 vs N1 vs N0)	1.48 [1.23–1.78]	<0.001***	1.86 [1.04–3.32]	0.037*
pM stage (M1 vs M0)	1.79 [1.00–3.22]	0.050*	NA	NA
RAD54L expression (high vs low)	1.85 [1.13–3.04]	0.014*	2.39 [1.24–4.61]	0.009**

**Notes:**

* *P* < 0.05.

** *P* < 0.01.

*** *P* < 0.001.

HR, Hazard Ratio; CI, Confidence Interval.

## Discussion

In this study, we identified upregulated HRR pathway and its key functional genes RAD54L in early-stage LUAD. GSEA and KEGG analysis indicated HRR pathway is up-regulated in T1 stage LUAD. Survival analysis of RAD54L expression in TCGA and validation cohort showed identical results that higher expression of RAD54L was associated with poor overall survival (*P* = 0.001, 0.002 respectively). Multivariate cox regression analysis in both TCGA and validation cohort indicated that RAD54L expression and pathological TNM staging are independent prognostic factors of overall survival in LUAD. From this retrospective study, we confirm that dysregulation of HRR pathway up-regulation of RAD54L could be detected in early-stage LUAD.

Previous research has shown that high-level expression of RAD51, another important functional gene in HRR pathway, was an independent prognostic marker of overall survival in NSCLC patients (Qiao et al., 2005). RAD54 is an important member of the SNF2/SWI2 protein family, and plays a significant role in the HRR pathway for double-strand DNA break repair, which interacts with RAD51 to facilitate the assembly and function of the Rad51 nucleoprotein filament and promote the strand invasion step for this double strand DNA repair process (Ceballos & Heyer, 2011; Goyal et al., 2018). In addition, the RAD54 gene is the key point in the procedure of strain invasion and the constitution of a D-loop structure, which functions to regulate DNA synthesis immediately (Crickard et al., 2018; Zafar et al., 2018). Consistent with the important role of RAD54 protein in DNA double strand break repair, gene mutations at this locus have been detected in a great number of human cancers, including non-Hodgkin’s lymphoma, colon adenocarcinoma and invasive ductal breast carcinoma (Liu et al., 2018; Mazin et al., 2010).

Association of DDR system with drug resistance and regulation of cytotoxic effect in chemotherapy and radiotherapy are widely studied. Radiotherapy and most cytotoxic drugs for lung cancer have been considered to act by inducing DNA damage, the response of which in tumor cells associates with the therapeutic effects (O’Connor, 2015). Combination of DDR-target drug with chemotherapy or radiotherapy improves overall sensitivity and overcome resistance to traditional DNA damage treatments (Thomas & Pommier, 2016).

In recent years, there has been a better understanding of mechanism of HRR pathway and the regulatory effect in chemotherapy, radiotherapy, and immunotherapy. Targets in the HRR pathway have reach with some initial success, which is one of essential pathways of DDR system. From this retrospective study, we hypothesized that tumor cells could resist external damage by utilizing the HRR pathway in LUAD, which could be suggested from other studies that HRR pathway would induce lung cancer cell resistance to chemotherapy (Zafar et al., 2018). Upregulation of HRR pathway and RAD54L expression in choroid plexus carcinoma to reduce replication stress and genotoxic damage for tumor progression (Tong et al., 2015). Deficiency of Rad54 protein in mouse embryonic stem cells damaged HRR-mediated DNA repair and enhanced the sensitivity of these cells to ionizing radiation and mitomycin C (Dronkert et al., 2000; Agarwal et al., 2011; Wesoly et al., 2006; Essers et al., 1997). HRR related gene panel, including RAD54L were performed in 60 patients with urothelial cancer treated with anti-PD-1/PD-L1, showing that patients harboring DDR deficiency has longer PFS and OS than wild-type DDR (Teo et al., 2018). Through analysis of solid tumor in TCGA and ICGC, co-mutation of HRR and other DDR pathways correlated to better clinical outcome with immune checkpoint inhibitors (Wang et al., 2018).

This study does have several limitations. Through biostatics analysis, we found that the HRR pathway may be one of functional pathways in tumorigenesis of early-stage LUAD and expression of RAD54L correlated with prognosis, which need to be validated in clinical situation by immunohistochemistry staining in biopsy or resection tissue. Data of treatment intervention in our study cohorts could not be obtained from the dataset, no conclusions could be drawn about a potential role of the HRR pathway and RAD54L as predictor of response to therapy. In the future, studies could be conducted to investigate specific molecular mechanisms of HRR pathway in LUAD, and explore more prognostic marker and therapeutic target.

## Conclusions

In conclusion, the HRR pathway was up-regulated in LUAD, among which RAD54L were most significant DEGs in T1 stage LUAD, indicating the up-regulation of DNA damage and repair system in the early stage of LUAD. The relatively lower expression of the differential gene RAD54L in the T1 stage LUAD showed better overall survival in our study, which suggested that this gene may be a putative biomarker in primitive LUAD. We also found that RAD54L is an independent prognostic factor for LUAD, which could be detected in early stage. Quantitative RAD54L sequencing and assessment may improve the prediction of clinical outcomes in early-stage LUAD. This study provided further insight into a potential mechanism of disease progression in T1 stage patients. We suggest that early-stage LUAD patients with higher expression of RAD54L, who may have a worse prognosis, require early postoperative adjuvant intervention.

## Supplemental Information

10.7717/peerj.10680/supp-1 Supplemental Information 1 Survival analysis of RAD54L in KM plot.

10.7717/peerj.10680/supp-2 Supplemental Information 2 Survival analysis of RAD54L in GEPIA.

## Additional Information and Declarations

### Data Availability

The following information was supplied regarding data availability: Raw data are available at The Cancer Genome Atlas (TCGA-LUAD: https://portal.gdc.cancer.gov/projects/TCGA-LUAD) and at NCBI GEO (GSE50081).
