# Supplementary figures and images for: Homologous recombination repair rathway and RAD54L in early-stage lung adenocarcinoma
Source: PeerJ. 2021 Feb 16;9:e10680. doi: 10.7717/peerj.10680 (PMC7894105; doi:10.7717/peerj.10680)

A

KM plot-LUAD

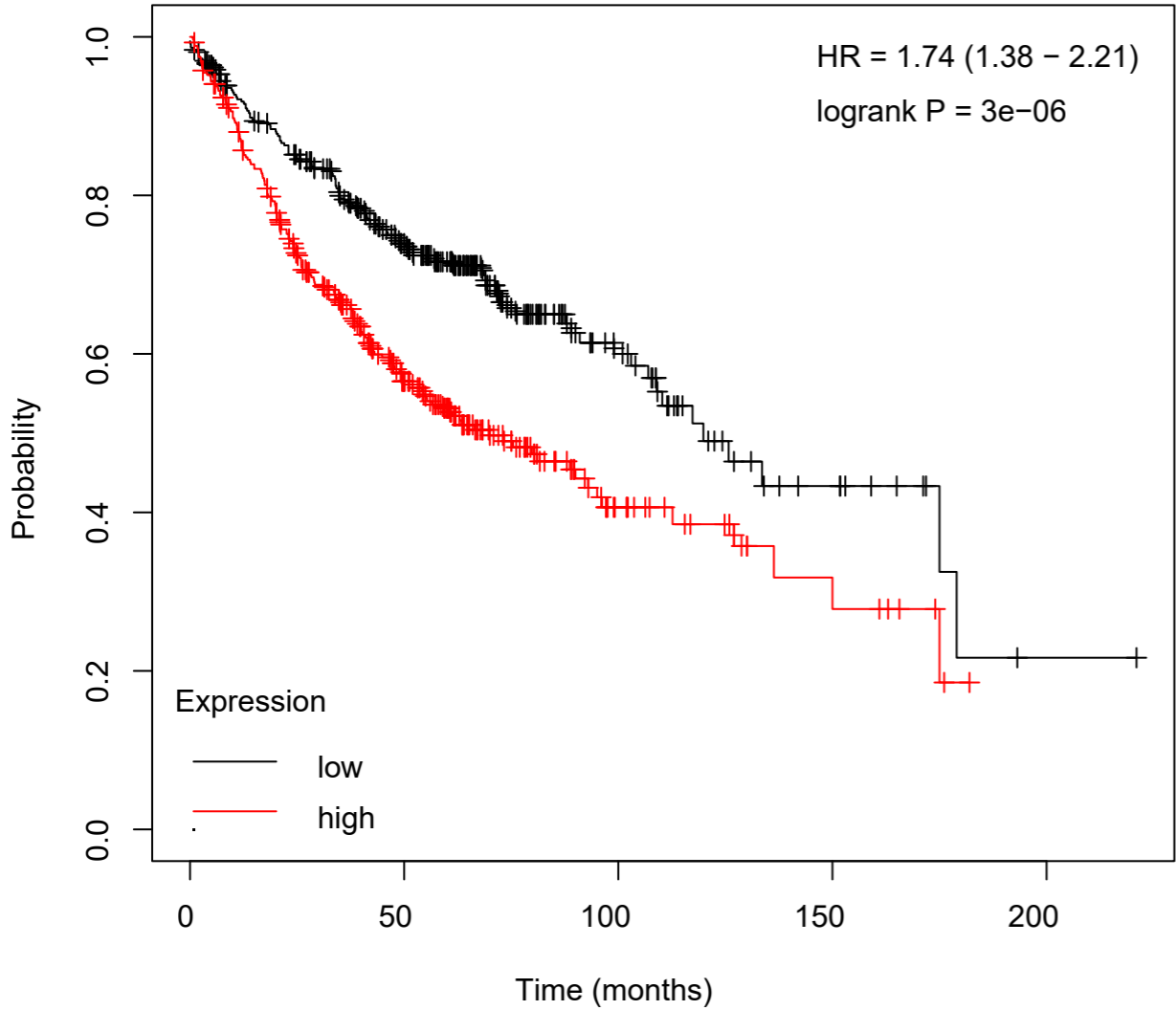

B

KM plot-GSE30219

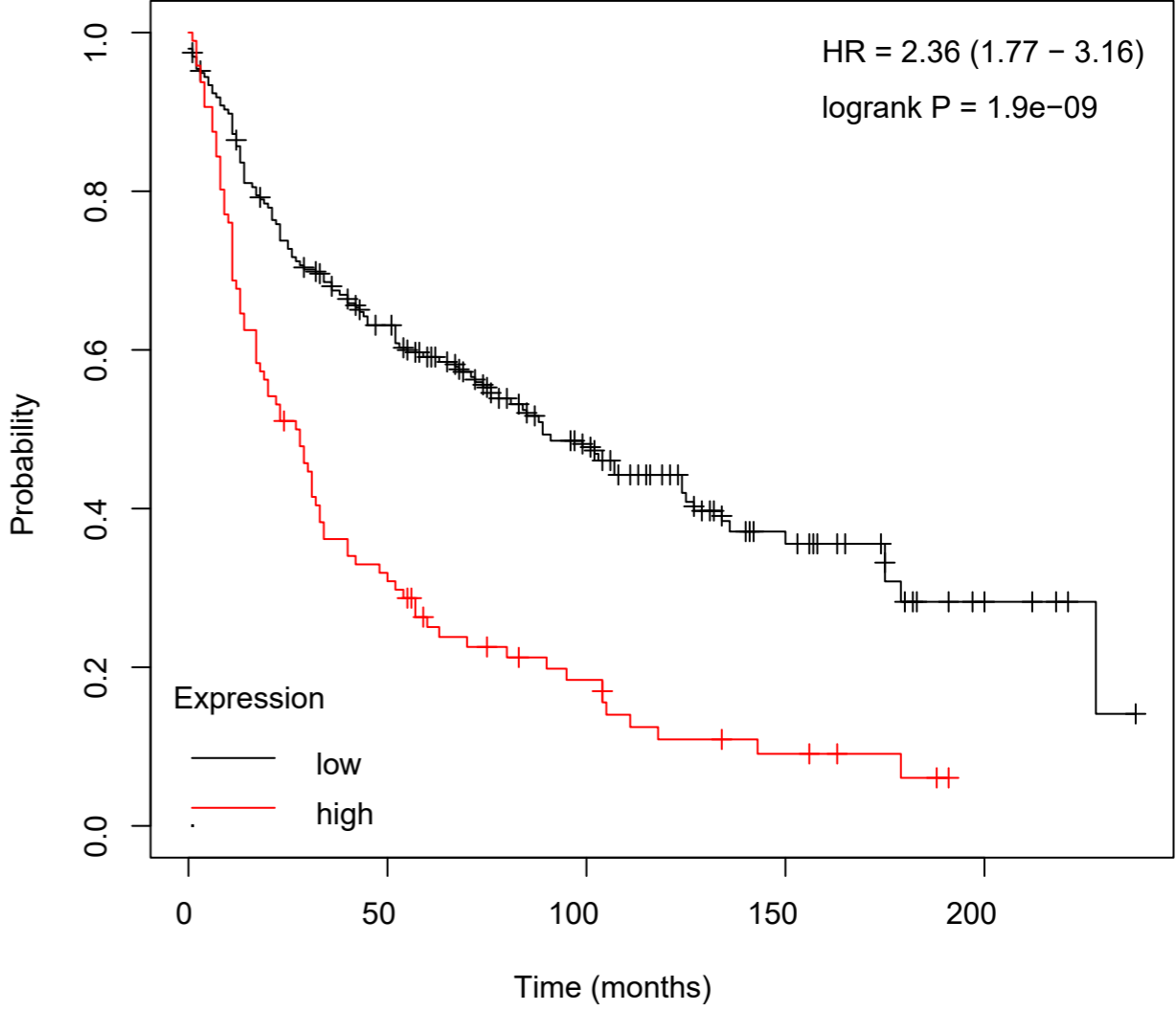

C

KM plot GSE37745

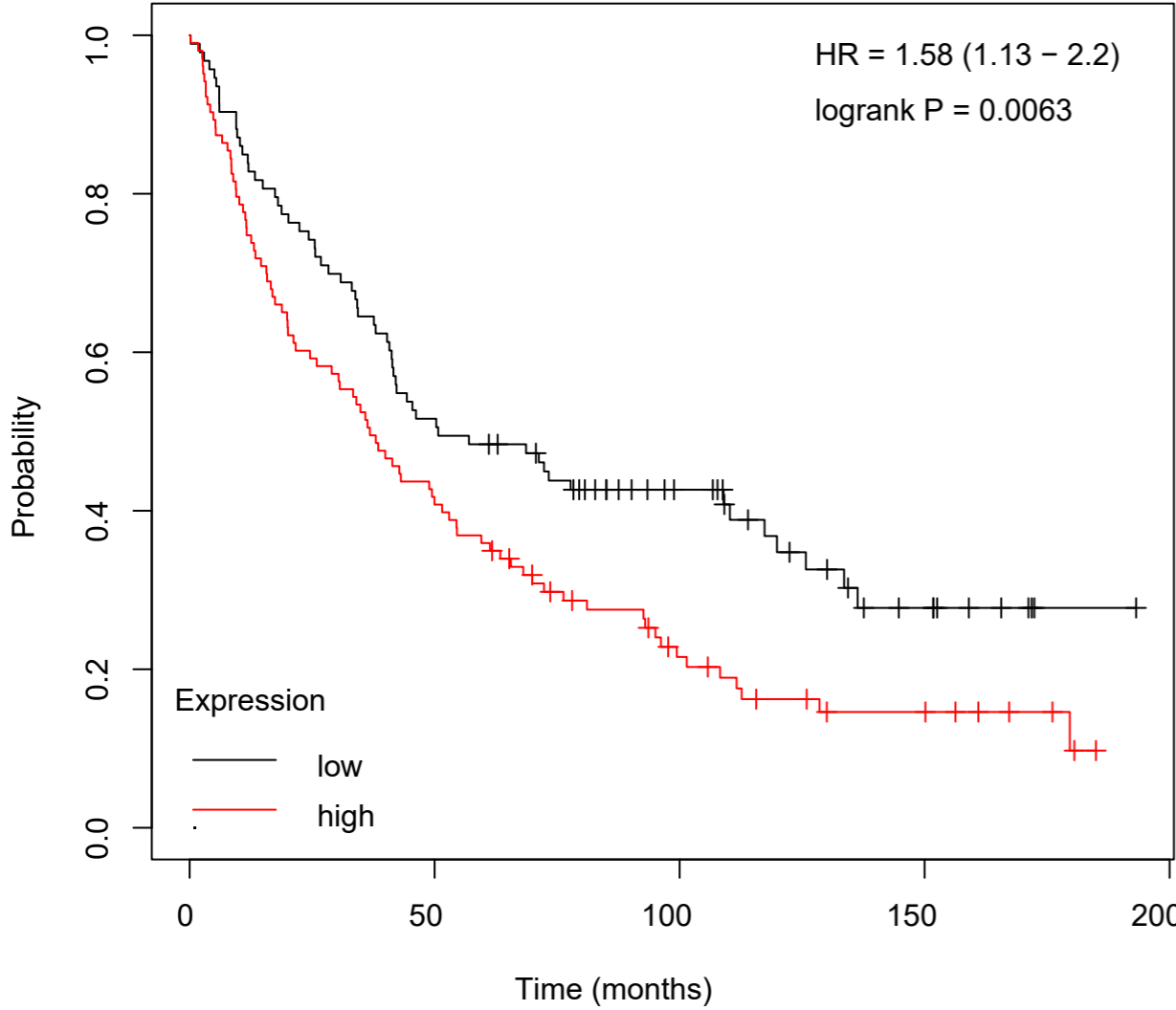

D

KM plot-GSE31210

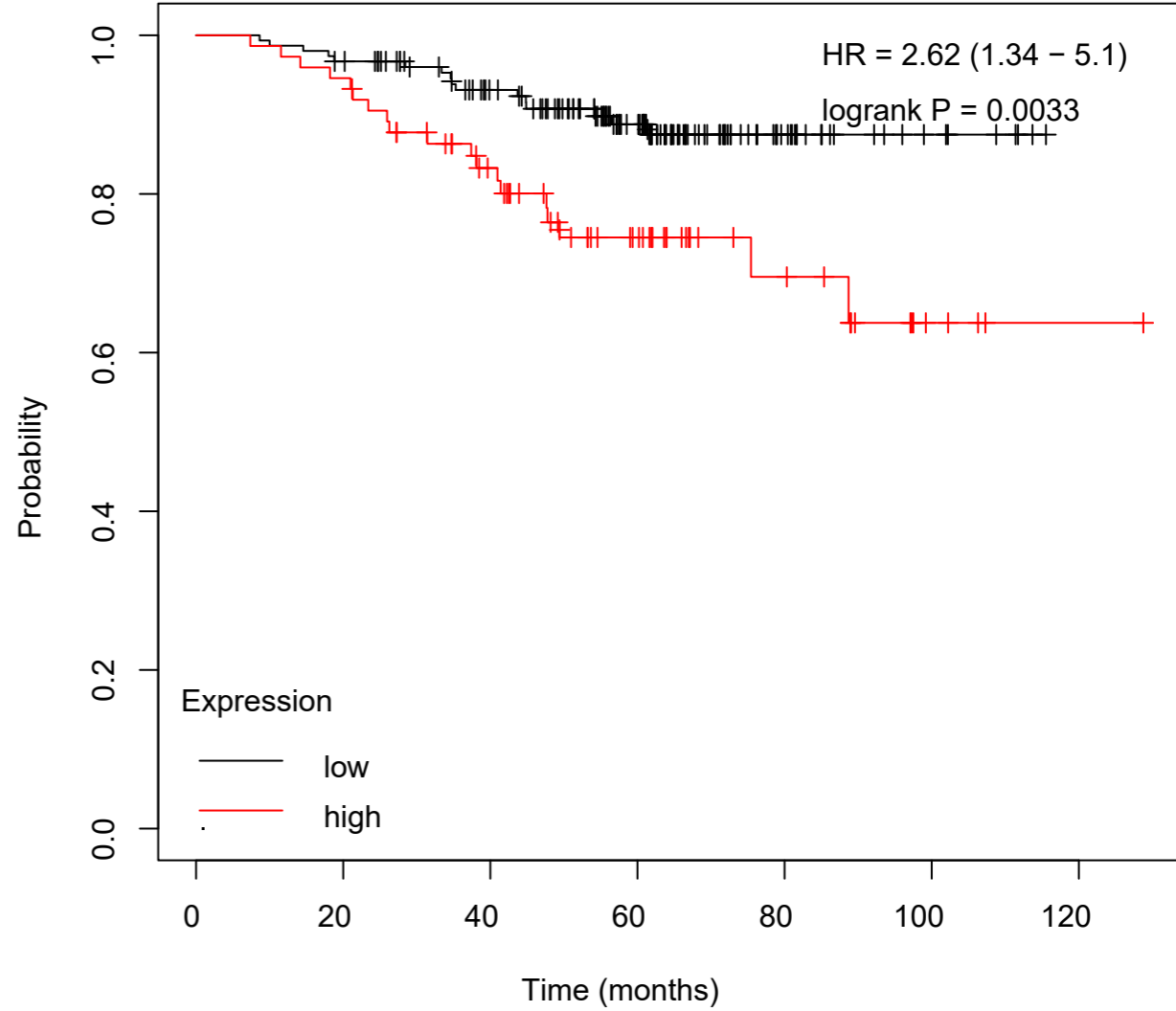

Supplement: Supplemental Information 1 [file peerj-09-10680-s001.pdf]

# Overall Survival

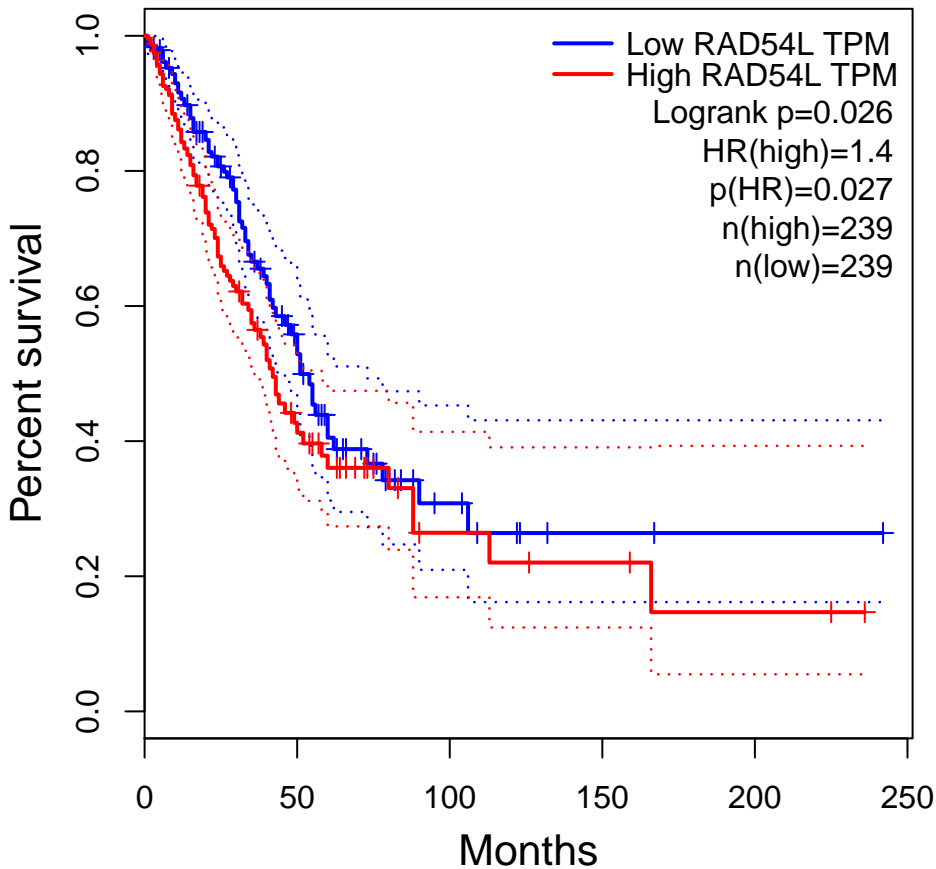

Supplement: Supplemental Information 2 [file peerj-09-10680-s002.pdf]
